# Concurrent Vulvar and Cervical Tuberculosis With Suspected Endometrial Involvement in a Postmenopausal Woman: A Highly Unusual Case Report From Guangzhou, China

**DOI:** 10.1002/ccr3.73187

**Published:** 2026-07-22

**Authors:** Yaping Chen, Hua Li, Pengyan Dong, Mingxing Liu, Yaokun Zeng, Qi Wang, Yu Yang

**Affiliations:** ^1^ State Key Laboratory of Respiratory Disease, Guangzhou Key Laboratory of Tuberculosis Research Guangzhou Chest Hospital, Institute of Tuberculosis, Guangzhou Medical University Guangzhou P.R. China; ^2^ The Third Affiliated Hospital of Guangzhou Medical University Guangzhou P.R. China

**Keywords:** case report, cervical tuberculosis, female genital tuberculosis, postmenopausal bleeding, suspected endometrial involvement, vulvar tuberculosis

## Abstract

Female genital tuberculosis should be considered in the differential diagnosis of postmenopausal women presenting with abnormal vaginal bleeding, even in the absence of pulmonary tuberculosis history. For confirmed vulvar and cervical lesions, biopsy with acid‐fast staining and culture enables prompt diagnosis and cure, while suspected endometrial involvement requires histopathological and microbiological confirmation.

## 1 Introduction

According to the WHO Global Tuberculosis Report [1], the number of newly diagnosed tuberculosis (TB) cases globally reached 10.7 million in 2024, with adult women accounting for 35%. As one of the most high‐burden country for TB, China has 34 million women at risk of female genital tuberculosis (FGTB), and the disease predominantly affects individuals aged 20–40 years [2]. Female genital tuberculosis represents a significant but under‐recognized form of extrapulmonary TB in China. Owing to its non‐specific clinical presentation, it is frequently misdiagnosed. In postmenopausal women, abnormal vaginal bleeding is typically attributed to malignancy or endocrine etiologies, while TB is rarely incorporated into the differential diagnostic framework [3]. The pathophysiology of FGTB in postmenopausal women remains incompletely understood. While estrogen decline has been associated with immune senescence in aging populations, direct evidence linking menopausal hormonal changes to increased susceptibility to genital TB is currently lacking. Nonetheless, despite its rarity, genital tuberculosis should be considered as a potential etiological factor, as it may present with symptoms including irregular bleeding, ulcerative lesions, and vaginal discharge [4]. This case report underscores the significance of including FGTB in the differential diagnosis of postmenopausal bleeding.

FGTB is a rare extrapulmonary manifestation of *Mycobacterium tuberculosis* infection, often misdiagnosed due to its nonspecific clinical presentation. The reproductive system predominantly affects women of childbearing age, with fallopian tubes being the most frequently involved organs (95%–100% of cases). In contrast, vulvar and vaginal involvement is markedly uncommon, representing only approximately 1%–2% of cases [5, 6]. This anatomical predilection may be attributed to several factors. First, the fallopian tubes possess an abundant vascular supply that facilitates hematogenous seeding of mycobacteria, whereas the vulvar squamous epithelium provides an effective barrier against mycobacterial invasion. Second, vulvar tuberculosis typically occurs secondary to hematogenous dissemination from a distant focus or direct extension from the upper genital tract, rather than as a primary infection. Sexual transmission as a primary route, while documented, remains exceptionally rare [6, 7, 8]. To the best of our knowledge, concurrent vulvar and cervical tuberculosis in a postmenopausal woman has been rarely reported in the literature, rendering this presentation highly unusual.

## 2 Case Presentation

A 63‐year‐old woman presented to Guangzhou Chest Hospital on September 18, 2023, with a 10‐month history of “irregular vaginal bleeding following menopause.” Over the preceding 10 months, she experienced recurrent episodes of light pink discharge lasting several days, which resolved spontaneously without associated abdominal pain. Similar self‐limiting episodes have been described in postmenopausal genital TB [3]. The patient did not seek medical attention for these symptoms. She was otherwise healthy and denied any history of tuberculosis or contact with infectious diseases. In 2022, she underwent right adnexectomy for pelvic inflammatory disease. Previous adnexal surgery is a recognized risk factor for re‐activation of latent genital TB [9]. She had been postmenopausal for 21 years with a gravidity of 5 and parity of 4 (G5P4A1), including four vaginal deliveries and one induced abortion.

### 2.1 Physical Examination

The patient was afebrile with a body temperature of 36.5°C, respiratory rate of 20 breaths per minute, pulse rate of 91 beats per minute, and blood pressure of 123/75 mmHg. She appeared in good general health with no oral ulcers or abnormalities detected on auscultation. Pulmonary examination revealed clear breath sounds bilaterally, and cardiac examination demonstrated regular rhythm without murmurs. Abdominal examination showed no tenderness or rebound tenderness, consistent with her stable clinical condition.

### 2.2 Gynecological Examination

**FIGURE 1:**
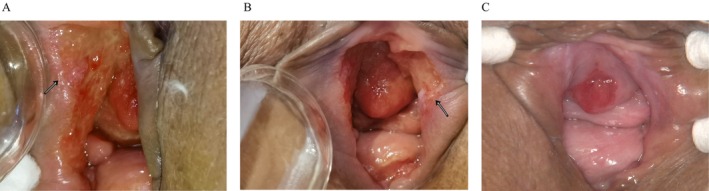
Vulvar tuberculosis before and after treatment. (A) Ulcer on the right labia minora with yellow discharge before treatment; (B) Ulcer on the left labia minora before treatment; (C) After anti‐tuberculosis treatment, the ulcers healed, and the mucosa became smooth.

**FIGURE 2:**
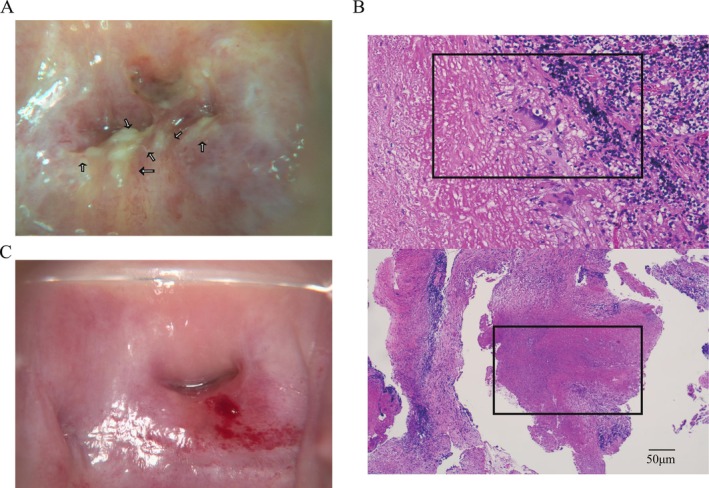
Clinical, pathological manifestations, and treatment response of cervical tuberculosis. (A) Yellow‐white granular lesions at the cervical os before treatment; (B) Cervical biopsy showing granulomatous inflammation with necrosis (acid‐fast staining suggesting tuberculosis; Scale bar: 50 μm; Original magnification: ×400); (C) After treatment, the cervical lesions disappeared, and the surface became smooth.

No cervical motion tenderness was elicited, and no masses were palpable in the uterus or adnexal regions. External genital examination revealed bilateral irregular ulcerative lesions on the labia minora with purulent discharge (Figure 1A,B). Gynecological examination demonstrated cervical hypertrophy with yellowish‐white granular lesions at the cervical os (Figure 2A).

### 2.3 Laboratory and Imaging Findings

Admission laboratory studies revealed a white blood cell count of 7.55 × 10^9^/L with 69.6% neutrophils and 21.1% lymphocytes, hemoglobin of 126 g/L, and platelet count of 250 × 10^9^/L. Liver function tests showed albumin of 40 g/L and alanine aminotransferase of 12.7 U/L. Inflammatory markers demonstrated an elevated erythrocyte sedimentation rate of 51 mm/h. γ‐interferon release assay was positive, with acid‐fast staining also positive. A positive interferon‐γ release assay and elevated acute‐phase reactants support active extrapulmonary TB [10]. Screening tests for vaginal discharge, human papillomavirus (HPV), and thin‐layer cytology (TCT) were unremarkable.

**FIGURE 3:**
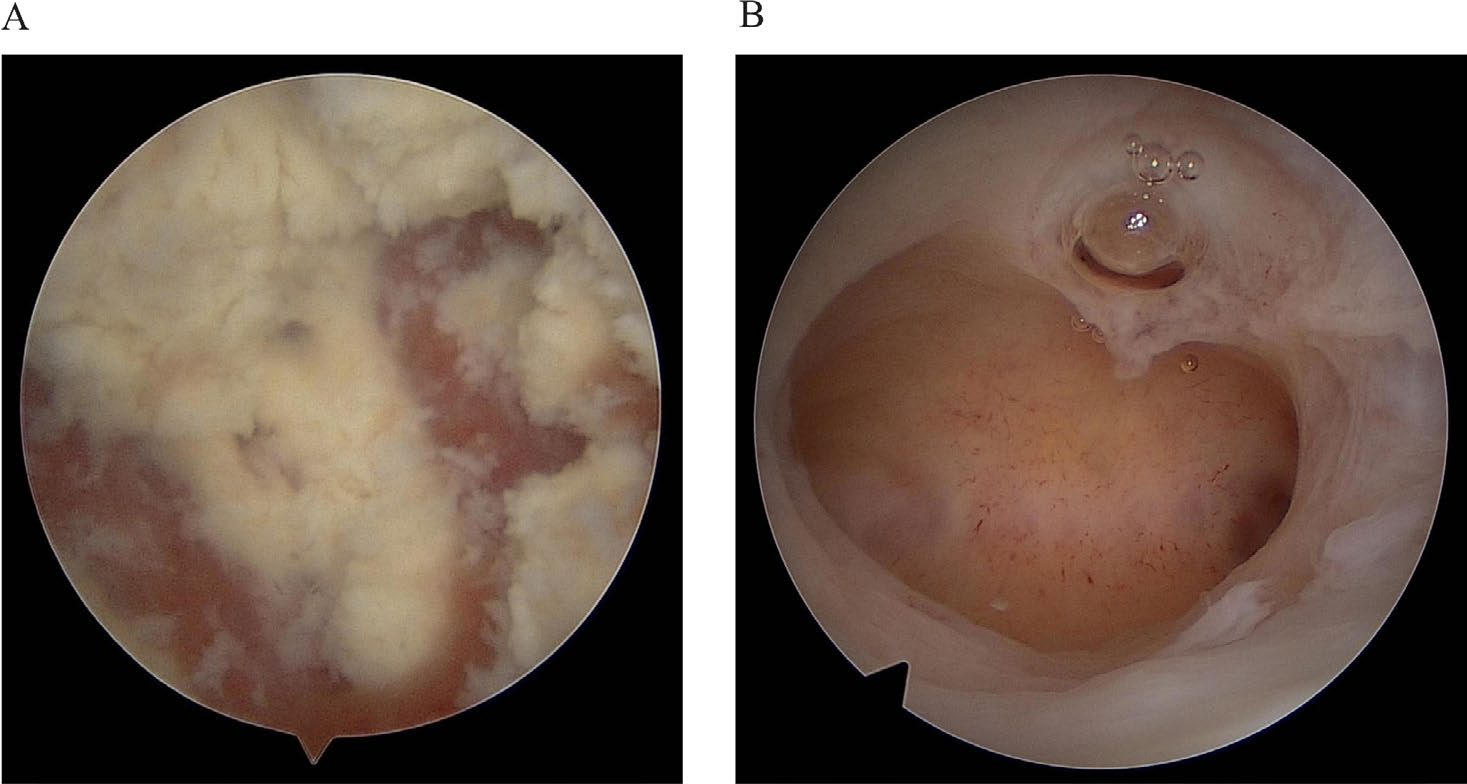
Hysteroscopic comparison. (A) Uterine cavity filled with yellow‐white flocculent material before treatment; (B) Uterine cavity restored to smoothness after 4 months of anti‐tuberculosis treatment.

Chest radiography was normal. Pelvic ultrasonography revealed a retroverted uterus with thickened endometrium measuring 26 mm. Endometrial thickness > 5 mm in postmenopausal women warrants tissue diagnosis to exclude both malignancy and TB [11]. Gynecological examination demonstrated bilateral labial ulcers with purulent discharge and cervical hypertrophy with yellowish‐white granular lesions (Figure 3A).

## 3 Methods

### 3.1 Differential Diagnosis

The primary differential diagnoses considered for this postmenopausal patient with vaginal bleeding and vulvar ulcers included:

Genital malignancy: Given the patient's postmenopausal status and endometrial thickening (26 mm), endometrial or cervical carcinoma was the primary concern. However, TCT and HPV screening were unremarkable, and histopathology did not reveal malignant cells.

Behçet's disease: Vulvar tuberculosis typically presents with ulcerative lesions and must be differentiated from Behçet's disease [12, 13]. This patient did not exhibit typical features of Behçet's disease (no oral ulcers, no ocular involvement, no other systemic manifestations).

Chronic pelvic inflammatory disease: Persistent vaginal discharge may suggest chronic pelvic inflammatory disease. However, given the patient's postmenopausal status, absence of active infection signs, and lack of abdominal tenderness, this was considered unlikely.

Hormonal/endocrine disorders: While hormonal fluctuations during menopause may cause bleeding, they do not explain the vulvar ulcers, cervical granular lesions, or positive microbiological findings.

### 3.2 Investigations

Diagnostic specimens were obtained from distinct anatomical sites with the following analyses:

Vulvar biopsy: Histopathological examination with hematoxylin–eosin staining demonstrated granulomatous inflammation with caseous necrosis. Acid‐fast staining revealed mycobacterial organisms (Figure 2B; Scale bar: 50 μm; Original magnification: ×400).

Cervical biopsy: Histopathological examination with hematoxylin–eosin staining demonstrated granulomatous inflammation with caseous necrosis. Caseating granulomata on hematoxylin‐eosin stain remain the histological gold standard for genital TB [5].

Vaginal discharge: Culture using Lowenstein‐Jensen medium demonstrated mycobacterial growth on October 19, 2023. Strain identification on October 30, 2023, confirmed *Mycobacterium tuberculosis* complex. Drug susceptibility testing revealed sensitivity to isoniazid, rifampicin, and ethambutol.

Endometrial cavity: Hysteroscopic evaluation revealed yellow‐white flocculent material filling the uterine cavity (Figure 3A). Endometrial tissue was sampled during the procedure; histopathological examination of the curettage specimen revealed necrotic tissue with extensive inflammatory cell infiltration. However, this endometrial sample was not subjected to acid‐fast staining, mycobacterial culture, or molecular testing.

### 3.3 Treatment

The patient was diagnosed with vulvar and cervical with suspected endometrial tuberculosis based on clinical, pathological, and microbiological findings. Anti‐tuberculosis treatment was initiated on September 25, 2023, consisting of daily oral isoniazid (0.3 g), rifampicin (0.45 g), pyrazinamide (1 g), ethambutol (0.75 g), and levofloxacin (0.5 g). The addition of levofloxacin was at the treating physician's discretion based on the following clinical considerations: the patient's extensive multi‐site genital involvement and severity of presentation; the endometrial histopathology showing necrotic tissue with extensive inflammatory cell infiltration, which raised the possibility of concurrent intrauterine infection and warranted broader antimicrobial coverage; and the need to reduce the risk of treatment failure in this complex case with atypical presentation and delayed diagnosis (10‐month history of symptoms). While a standard four‐drug regimen is recommended for drug‐susceptible extrapulmonary TB per WHO guidelines, fluoroquinolones remain important reserve agents that may be employed in complex cases based on clinical judgment [14]. The patient demonstrated excellent clinical and microbiological response to this regimen.

## 4 Conclusion and Results

Following treatment initiation, the patient demonstrated significant clinical improvement with resolution of vulvar ulcers (Figure 1C), cessation of abnormal vaginal bleeding, and normalization of discharge. The cervical lesions disappeared, and the surface became smooth (Figure 2C).

Three‐month follow‐up culture of vaginal discharge showed no mycobacterial growth. Hysteroscopic evaluation after 4 months of anti‐tuberculosis treatment showed the uterine cavity restored to smoothness (Figure 3B). Six‐month follow‐up confirmed complete clinical and microbiological cure. This case underscores the need for heightened clinical suspicion of FGTB in postmenopausal women presenting with abnormal vaginal bleeding. Early tissue diagnosis and appropriate anti‐tubercular therapy can prevent unnecessary interventions and ensure complete recovery. We acknowledge that endometrial tissue sampling for histopathological and microbiological confirmation was not performed, representing a limitation of this case report.

## 5 Discussion

Tuberculosis of the reproductive system, although uncommon, represents a significant public health challenge in TB‐endemic regions. As detailed in the Introduction, FGTB demonstrates a marked anatomical predilection for the fallopian tubes, while vulvar and vaginal involvement is markedly uncommon. To contextualize the rarity of this presentation, we conducted a literature search of PubMed/Medline, CNKI, and Wanfang databases and identified limited reported cases of concurrent vulvar and cervical tuberculosis in postmenopausal women. The specific combination of vulvar, cervical, and suspected endometrial involvement in a postmenopausal patient without prior pulmonary TB history appears to be exceedingly uncommon. This case report presents an atypical manifestation of tuberculosis in a postmenopausal woman, emphasizing the critical importance of prompt diagnosis and treatment.

The patient experienced irregular vaginal bleeding for 10 months without prior history of pulmonary tuberculosis. Positive interferon‐gamma release assay and acid‐fast staining suggested latent *M. tuberculosis* infection, with vaginal discharge culture confirming *M. tuberculosis* . Based on comprehensive clinical and pathological findings, the diagnosis of concurrent vulvar, cervical tuberculosis with suspected endometrial involvement was established. Standard anti‐tuberculosis treatment resulted in significant symptomatic improvement and clear therapeutic benefit.

Tuberculosis in postmenopausal women is frequently under‐recognized in medical literature. When these patients present with vaginal bleeding, potential etiologies include hormonal changes and neoplastic processes. The pathophysiology of FGTB in postmenopausal women remains incompletely understood. While estrogen decline has been associated with immune senescence in aging populations, direct evidence linking menopausal hormonal changes to increased susceptibility to genital TB is currently lacking. In this case, while cancer screening effectively excluded malignancy, pathological examination revealed granulomatous inflammation with necrosis, ultimately confirming tuberculosis.

Persistent vaginal discharge, often watery or purulent with potential malodor, may suggest chronic pelvic inflammatory disease, potentially leading to misdiagnosis. Although this patient had a 10‐month history of irregular vaginal bleeding, significant abnormal discharge was not prominent. However, gynecological examination revealed labial ulcers with purulent discharge and thick white cervical discharge. Given the patient's postmenopausal status and absence of active infection signs, pelvic inflammatory disease was considered unlikely.

Vulvar tuberculosis typically presents with ulcerative lesions and must be differentiated from Behçet's disease [12, 13]. This patient did not exhibit typical features of Behçet's disease, and her condition improved significantly with anti‐tuberculosis therapy, making this diagnosis improbable. Although systemic symptoms were absent, the diagnosis of genital tuberculosis was confirmed through comprehensive evaluation.

Concurrent vulvar and cervical tuberculosis in a postmenopausal woman is highly unusual. To the best of our knowledge, this specific combination in a postmenopausal patient has been rarely reported. This case illustrates the complexity of postmenopausal vaginal bleeding and emphasizes the importance of considering tuberculosis as a potential underlying etiology.

We acknowledge several limitations of this case report. First, endometrial tuberculosis was diagnosed clinically based on hysteroscopic findings and treatment response, but was not confirmed by endometrial biopsy, histopathology, acid‐fast staining, culture, or molecular testing. This represents the most significant limitation of our study. Second, the addition of levofloxacin to the standard regimen, while clinically justified by the treating team, deviates from standard WHO recommendations for drug‐susceptible extrapulmonary TB. Third, the rarity claim of this presentation, while supported by our literature search, would benefit from systematic review methodology.

Clinicians should include tuberculosis in their differential diagnosis, and future research should focus on developing specific treatment protocols for vulvar and cervical tuberculosis to improve clinical outcomes.

## Author Contributions

**Hua Li:** data curation, investigation, writing – review and editing. **Yu Yang:** writing – review and editing, formal analysis, funding acquisition, conceptualization, methodology, project administration, resources, supervision. **Pengyan Dong:** resources, writing – review and editing, data curation, investigation. **Yaping Chen:** writing – original draft, investigation, formal analysis, conceptualization, data curation, visualization, writing – review and editing. **Qi Wang:** writing – review and editing, supervision, funding acquisition. **Mingxing Liu:** writing – review and editing, resources. **Yaokun Zeng:** resources, investigation, writing – review and editing.

## Funding

This work was supported by the Guangdong Basic and Applied Basic Research Foundation of Natural Science Foundation (Grant 2023A1515010461), Guangzhou Basic and Applied Basic Research Foundation of Natural Science Foundation (Grant 2024A03J0584), Guangdong Bureau of Traditional Chinese Medicine Scientific Research Project (Grants 20251291 and 20251290), Guangzhou Science and Technology Planning Project (Grant 2025A03J3606), Guangzhou Health Science and Technology Project (Grant 20242A011017) and Major scientific and technological projects of traditional Chinese medicine in Guangzhou (Grant 2025CX019).

## Ethics Statement

Guangzhou Chest Hospital granted ethical approval for this study [No. 2023(20)], and the participant provided written informed consent.

## Consent

Written informed consent was obtained from the patient for publication of this case report and accompanying images.

## Conflicts of Interest

The authors declare no conflicts of interest.

## Data Availability

All data are contained within the manuscript. The datasets used and analyzed during the current study are available from the corresponding author upon reasonable request.
